# FKBPL and its peptide derivatives inhibit endocrine therapy resistant cancer stem cells and breast cancer metastasis by downregulating DLL4 and Notch4

**DOI:** 10.1186/s12885-019-5500-0

**Published:** 2019-04-11

**Authors:** Lana McClements, Stephanie Annett, Anita Yakkundi, Martin O’Rourke, Andrea Valentine, Nermeen Moustafa, Abdelrahim Alqudah, Bruno M. Simões, Fiona Furlong, Amy Short, Stuart A. McIntosh, Helen O. McCarthy, Robert B. Clarke, Tracy Robson

**Affiliations:** 1 Centre for Experimental Medicine, School of Medicine, Dentistry and Biomedical Sciences, Queen’s University Belfast, Belfast, UK; 2 School of Pharmacy, Queen’s University Belfast, Belfast, UK; 3 The School of Life Sciences, University of Technology Sydney, Sydney, Australia; 4 Department of Molecular and Cellular Therapeutics, Irish Centre for Vascular Biology, Royal College of Surgeons in Ireland, RCSI, Dublin, Ireland; 5 Charles River Labs, 8-9 Spire Green Centre, Essex, Harlow, CM19 5TR UK; 6 School of Pharmacy, Hashemite University, Amman, Jordan; 7 Manchester Breast Centre, Division of Cancer Sciences, University of Manchester, Oglesby Cancer Research Building, Manchester, UK; 8 Centre for Cancer Research and Cell Biology, Queen’s University Belfast and Breast Surgery Department, Belfast City Hospital, Belfast, UK

**Keywords:** Metastasis, Triple negative breast cancer, Estrogen receptor, Endocrine therapy, Breast cancer stem cells, FKBPL, ALM201, AD-01, Notch4, DLL4, Tamoxifen, Letrozole

## Abstract

**Background:**

Optimising breast cancer treatment remains a challenge. Resistance to therapy is a major problem in both ER- and ER+ breast cancer. Tumour recurrence after chemotherapy and/or targeted therapy leads to more aggressive tumours with enhanced metastatic ability. Self-renewing cancer stem cells (CSCs) have been implicated in treatment resistance, recurrence and the development of metastatic disease.

**Methods:**

In this study, we utilised in vitro, in vivo and *ex vivo* breast cancer models using ER+ MCF-7 and ER- MDA-MB-231 cells, as well as solid and metastatic breast cancer patient samples, to interrogate the effects of FKBPL and its peptide therapeutics on metastasis, endocrine therapy resistant CSCs and DLL4 and Notch4 expression. The effects of FKBPL overexpression or peptide treatment were assessed using a t-test or one-way ANOVA with Dunnett’s multiple comparison test.

**Results:**

We demonstrated that FKBPL overexpression or treatment with FKBPL-based therapeutics (AD-01, pre-clinical peptide /ALM201, clinical peptide) inhibit i) CSCs in both ER+ and ER- breast cancer, ii) cancer metastasis in a triple negative breast cancer metastasis model and iii) endocrine therapy resistant CSCs in ER+ breast cancer, via modulation of the DLL4 and Notch4 protein and/or mRNA expression. AD-01 was effective at reducing triple negative MDA-MB-231 breast cancer cell migration (*n* ≥ 3, *p* < 0.05) and invasion (*n* ≥ 3, *p* < 0.001) and this was translated in vivo where AD-01 inhibited breast cancer metastasis in MDA-MB-231-lucD3H1 in vivo model (*p* < 0.05). In ER+ MCF-7 cells and primary breast tumour samples, we demonstrated that ALM201 inhibits endocrine therapy resistant mammospheres, representative of CSC content (*n* ≥ 3, *p* < 0.05). Whilst an in vivo limiting dilution assay, using SCID mice, demonstrated that ALM201 alone or in combination with tamoxifen was very effective at delaying tumour recurrence by 12 (*p* < 0.05) or 21 days (*p* < 0.001), respectively, by reducing the number of CSCs. The potential mechanism of action, in addition to CD44, involves downregulation of DLL4 and Notch4.

**Conclusion:**

This study demonstrates, for the first time, the pre-clinical activity of novel systemic anti-cancer therapeutic peptides, ALM201 and AD-01, in the metastatic setting, and highlights their impact on endocrine therapy resistant CSCs; both areas of unmet clinical need.

**Electronic supplementary material:**

The online version of this article (10.1186/s12885-019-5500-0) contains supplementary material, which is available to authorized users.

## Background

Breast cancer is a highly heterogeneous disease with subtypes based on hormone receptors, oestrogen or progesterone receptors (ER/PR) and HER2 overexpression. More recently, gene expression profiling led to identification of five main molecular subtypes of breast cancer: HER2 overexpression (ER−/PR−/HER2+), basal-like (ER−/PR−/HER2−/basal marker+), luminal A (ER+/PR+/HER2−/KI67-), luminal B (ER+/PR+/HER2−/KI67+ or ER+/PR+/HER2+/KI67+) and normal-like (ER+/PR+/HER2−/KI67-) [1]. Further subtypes have also been identified based on integrative analysis of gene expression and copy number, suggesting increased complexity of breast cancer heterogeneity [2]. Despite major breakthroughs in the treatment of breast cancer over the last twenty years, there is still a significant number of patients who do not respond, develop resistance to therapy, or experience tumour recurrence; late relapse in ER+ breast cancer continues to be a particular issue. There is now a plethora of evidence to suggest that cancer stem cells (CSCs) are responsible for the incidence of metastatic disease which is the main cause of death in patients with breast cancer [3]. Triple negative breast cancer or basal-like subtype constitutes around 20% of breast cancer cases and it is highly metastatic with limited therapeutic options [4]. Chemotherapy remains the only treatment option for this disease subtype. The chemotherapy resistant CSC population has increased metastatic potential in triple negative breast cancer through activation of oncogenic pathways such as STAT3, therefore there is an urgent need for new therapeutic options which target CSCs [5, 6]. On the other hand, the most common type of breast cancer, ER+ (luminal A/B or normal-like), is treated with endocrine therapy in both the adjuvant and metastatic settings [7]. Tumour recurrence in endocrine-resistant breast cancer patients leads to a more aggressive type of breast cancer with enhanced metastatic ability [8]. In patients treated with neoadjuvant letrozole, CD44^+^/CD24^−^ mammosphere forming cells, representative of CSCs, were increased and the remaining tumour cells appeared to have a mesenchymal phenotype consistent with the more aggressive, basal-like type of breast cancer [9]. This acquired endocrine therapy resistance has been attributed to the activation of survival pathways such as the epidermal growth factor receptor (EGFR) pathway and, more recently, the Notch pathway [10]. The Notch 4 receptor, in particular, regulates breast CSC activity [11] and it is also implicated in endocrine therapy resistance in women treated with tamoxifen [12, 13]. Furthermore, tumour and plasma levels of the Notch 1, 2, or 4 receptors and DLL4 ligand were positively correlated with nodal and distant metastases in breast cancer and shorter disease-free or overall survival compared to patients with high DLL4 levels [14, 15]. In relevant in vitro and in vivo cancer models, DLL4 has also been implicated in chemoresistance [16], tumour angiogenesis [17] and CSC activity [18]. Therefore, all of these studies suggest that DLL4 and Notch 4 are viable therapeutic targets for both triple negative and ER+ breast cancer treatment.

FK506-binding protein like (FKBPL) is a novel anti-tumour protein that belongs to the family of immunophilins, but is a divergent member lacking peptidyl prolyl isomerase activity [19]. Immunophilins orchestrate protein-protein interactions therefore regulating many cellular processes including cell signalling, differentiation, cell cycle progression, metabolic activity and apoptosis [20]. FKBPL has diverse anti-tumour roles both as an intracellular and extracellular protein. Intracellular FKBPL regulates ER signalling and, as such, has prognostic value in terms of breast cancer survival. This was demonstrated using publically available datasets [21] and in a meta-analysis of five independent breast tissue microarray (TMA) cohorts [21, 22]. In this cohort of 3277 patients, FKBPL was a significant and independent predictor of breast cancer specific survival (BCSS), with low FKBPL expression being associated with shorter BCSS (HR = 1.31, 95% CI 1.15–1.50, *p* < 0.001). Likewise, in a cohort of 2365 ER+ breast cancer patients, low FKBPL expression had also a significantly shorter BCSS compared to high FKBPL expression (HR = 1.34, 95% CI 1.13–1.58, *p* < 0.001) [21]. Similarly, RBCK1, an E3 ubiquitin-protein ligase, which regulates FKBPL levels, also demonstrated a potential role as a prognostic and predictive biomarker of response to endocrine therapy in breast cancer patients in terms of BCSS [23].

In addition to this intracellular role, FKBPL’s extracellular anti-angiogenic and anti-CSC roles were identified, potentially through its ability to target CD44 [5, 24, 25]. Upregulation of CD44 is associated with angiogenesis, stemness, tumourigenicity and cell migration [26]. The ‘first-in-class’ FKBPL-based peptides, AD-01 (24-amino acid pre-clinical therapeutic candidate) and ALM201 (23-amino acid clinical therapeutic candidate which has successfully completed a Phase Ia clinical trial [EudraCT 2014–001175-31]) [27], have also demonstrated strong anti-angiogenic and anti-CSC effects [5, 24, 25]. The anti-CSC activity of AD-01 led to downregulation of stem cell markers, Nanog, Oct4 and Sox2 in breast cancer cell lines while the intratumoural knockdown of FKBPL in a ZR-75 breast cancer xenograft mouse model increased the expression of Nanog/, Oct4 and Sox2 [5]; Sox2 has been implicated in both metastasis and endocrine therapy resistance [28–30]. Therefore, since FKBPL and its peptides have demonstrated inhibitory effects on angiogenesis [24, 25], CSC signalling [5] and ER signalling [21, 22], we hypothesised that FKBPL could also inhibit metastasis and endocrine therapy resistance driven by CSCs in breast cancer. Here, we show for the first time, that FKBPL and its therapeutic peptides reduce metastatic burden in a triple negative breast cancer model and inhibit endocrine therapy resistant CSCs, thereby reducing tumour initiation, in ER+ disease. Furthermore, we elucidate additional targets of FKBPL such as DLL4 and Notch 4, which in addition to CD44, are potentially involved in the multiple anti-tumour effects of FKBPL and its therapeutic peptide derivatives.

## Methods

### Cell culture

All cells were obtained from the American Type Culture Collection, authenticated by short-tandem repeat (STR) profiling carried out by the suppliers, and verified as mycoplasma-free. MDA-MB-231 CD44 stable knockdown (KD) cells were a gift from Prof. David Waugh (QUB) [31]. The MCF-7 and MDA-MB-231 cell lines were cultured in Dulbecco’s modified Eagle medium (DMEM; Life Technologies, UK) supplemented with 10% foetal calf serum (FCS; GE Healthcare, UK). Cells stably overexpressing FKBPL (D2 from parental cell line, MCF-7, and A3 from parental cell line, MDA-MB-231) were selected using 750 μg/mL G418 (Sigma, UK) and grown in the presence of 375 μg/mL (3.1D2) or 750 μg/mL (A3) G418 (Sigma, UK) as previously described [5]. Cell culture experiments were carried out at 37°C in a humidified atmosphere of 95% O_2_/5% CO_2_.

### Boyden chamber assays

A Boyden chamber assay was used to examine cell migration and invasion. MDA-MB-231 cells were treated with AD-01 (1 nM) for 24 h. Following 24 h cells were trypsinized and re-suspended, (1.0 × 10^4^ cells in 200 μl RPMI-1640 medium) and then placed into the uncoated (for migration) or Matrigel coated (for invasion) upper chambers (8-mm pore size; Millipore, USA). The lower chambers were filled with 600 μl complete medium with 10% FBS. After incubation for 12 h at 37 °C, non-invading cells were removed from the top of the chamber with a cotton swab. The invaded cells on the lower surface of the inserts were fixed and stained with 0.1% crystal violet, and five random fields for each insert were counted at 200× magnification.

### Primary samples

Solid breast tumour mastectomy samples or core biopsies treated in the neoadjuvant setting with letrozole were collected from patients with fully informed consent (NIB14–0117; Northern Ireland Biobank), cut into small pieces (1 mm), and digested for 2 h on a rotating platform in RPMI (Gibco, UK) containing 10% collagenase/hyaluronidase (Stem Cell Technologies, UK). Following tissue digestion, filtration through 70 μm and 40 μm cell strainers (BD Technologies, UK) was carried out and 500 cancer cells per cm^2^ were seeded in the mammosphere medium DMEM-F12 (Gibco, UK), containing B27 minus vitamin A (Life Technologies, UK), 20 ng/ml EGF (Roche, UK), PenStrep (Invitrogen, UK) ± ALM201 (100 nM) as previously described [5]. Frozen pleural effusion samples collected from the patients with metastatic breast (*n* = 3) with fully informed consent (COREC# 05/ Q1403/25 and 05/Q1403/159; Division of Cancer Sciences, Manchester, United Kingdom) were defrosted, cells counted and seeded in the mammosphere assay for 72 h ± ALM201 (100 nM) as previously described [5].

### Treatments

1.2 × 10^4^ MCF-7 cells were plated in a monolayer in complete medium for 24 h. The medium was replaced by DMEM-F12 containing 10% charcoal-stripped serum medium and 17β-estradiol (100 nM; Sigma, UK) was added to all wells except for the control well. Tamoxifen (1 μM; Sigma, UK) and ALM201 (1 nM) were added alone or in combination for 72 h and cells incubated at 37°C in a humidified atmosphere of 95% O_2_/5% CO_2_. In a separate experiment, MCF-7 and MDA-MB-231 cell monolayers were treated with AD-01 (100 nM) or ALM201 (100 nM) before being used in mammosphere assays, western blotting or quantitative real-time polymerase chain reaction (qRT-PCR).

### Mammosphere assay

A single cell suspension was prepared following enzymatic (0.125% Trypsin-EDTA (Invitrogen, UK)) and manual disaggregation and 500 cells/cm^2^ were seeded in low adherent culture 6-well plates (VWR, UK) coated with 1.2% poly-HEMA (Sigma-Aldrich, UK) in mammosphere medium at 37°C in a humidified atmosphere of 95% O2/5% CO_2_ for 5–7 days as described previously [5].

### Flow cytometry

MCF-7 and MDA-MB-231 were grown in a cell monolayer or as mammospheres for 72 h before cells were disaggregated and incubated with pre-conjugated primary antibodies BEREP4-FITC (1:10; Dako), CD44-APC (1:20; BD Pharmigen), and CD24-PE (1:10; Beckman Coulter) as previously described [11]. Fluorescence was measured using BD FACSCanto II and analyzed by WinMDI 2.9.

### Clonogenic assay

MCF-7, 3.1D2, MDA-MB-231 and A3 cells were plated at a density of 50 or 100 cells/cm^2^ per well in a six well plate containing DMEM + 10% FCS medium and incubated for 10 days at 37°C in a humidified atmosphere of 95% O_2_/5% CO_2_. Following incubation the medium was removed, colonies were fixed with 1% crystal violet/70% ethanol and holoclones/meroclones/paraclones counted manually.

### Western blotting

MDA-MB-231 or MCF-7 cells were treated with ALM201 or AD-01 (100 nM) for 24 h before cells lysates were prepared using Laemmli buffer (Sigma, UK) and subjected to western blotting as reported previously [25]. Primary antibodies used included: DLL4 (Abcam, UK, cat: ab7280; 1:500), Notch4-ICD (Abcam, UK, cat:ab33163; 1:400), FKBPL (Proteintech, USA cat: 10060–1-AP; 1:1,000), CD44H (R&D Systems, USA, cat: BBA10; 1:1,000), GAPDH (Sigma, UK; cat: G9545; 1:10,000). HRP-linked secondary antibodies were either anti-mouse or anti-rabbit (GE Healthcare, UK; 1:10,000). Densitometry was performed using ImageJ software (NIH, USA) and adjusted to GAPDH.

### Quantitative real-time PCR

Following treatment of the adherent cells, as described above, RNA was extracted using GeneJET RNA purification kit (Fisher Scientific, UK) according to manufacturer’s instructions and RNA was quantified using a Nanodrop spectrophotometer (Thermo Fisher Scientific, Basingstoke, UK). Complimentary DNA (cDNA) was produced using Transcriptor first stand cDNA synthesis kit (Roche, Herefordshire, UK) according to manufacturer’s instructions. qRT-PCR was performed using the Lightcycler 480 PCR machine (Roche, UK). All Taqman primer probe sets were supplied by Roche (DLL4, cat:100073803; GAPDH, cat: 100065048; β-Actin, cat: 100063228).

### In vivo lung metastasis assay

In one set of experiment, 8–12 week old in-house bred female SCID mice (C.B-17/IcrHsd-Prkdc^scid^Lyst^bg^) were selected at random and pre-treated subcutaneously (s/c) once daily (a.m.) with AD-01 (0.003 or 0.3 mg/kg/day, *n* = 5) or PBS (*n* = 6) for one week prior to injection with 5 × 10^5^ MDA-MB-231-lucD3H1 cells, followed by continuation of treatment with AD-01 or PBS for a further 28 days. Lung cell load was assessed following i.p. injection of luciferin (150 mg/kg) on day 0 when mice were inoculated with cells, then lung metastatic colonization was assessed at day 28,), using non-invasive bioluminescence of total photon flux. In the second experiment, MDA-MB-231-LucD3H1 cells were grown in a monolayer and treated with AD-01 (1 nM) for 1 day before 8–12 week old female SCID mice were inoculated intravenously with 4 × 10^5^ pre-treated or mock (PBS) treated MDA-MB-231-LucD3H1 cells. Following inoculation, mice with detectable lung metastasis deposits were treated with control (PBS, *n* = 5) or AD-01 (0.3 mg/kg/day, *n* = 5 and 0.003 mg/kg/day, *n* = 5) for 26 days via i.p. injection. On day 26, primary experimental outcome i.e. lung metastatic colonization was assessed using non-invasive bioluminescence of total photon flux. At the end of the experiment, mice were euthanized by the carbon dioxide method. One-way ANOVA with post-hoc Dunnett’s multiple comparisons statistical test was used to compare the metastatic burden between control and the two treatment mice groups. All animals were of a similar weight (approx. 20 g) at the start of the experiments; weight and animal wellbeing was monitored at least twice weekly. Mice were housed in a group of up to 5 per cage in special SPF cages which included autoclaved bedding material. All in vivo procedures were carried out at the Biological Resource Unit at Queen’s University Belfast.

### Limiting dilution in vivo assay

MCF-7 cells (5 × 10^6^) were implanted intradermally into 8–12 week old in-house bred female SCID mice bearing oestrogen pellets (0.25 mg). Once MCF-7 xenografts were established (100–150 mm^3^), the following treatments were administered to randomly selected mice once daily (a.m.): 1) vehicle control via oral gavage (100μl) and PBS s/c (100 μl; *n* = 6), 2) tamoxifen citrate (Sigma, Cambridge, UK) via oral gavage (250 μg/100 μl; *n* = 4), 3) ALM201 s/c (0.3 mg/kg/day; *n* = 4) and 4) tamoxifen citrate via oral gavage (250 μg/100 μl) and ALM201 s/c (0.3 mg/kg/day; *n* = 4). The treatments were administered for the duration of 21 days and tumours were measured every 3 days. Following three weeks of treatment, mice were euthanized using the carbon dioxide method, tumours were excised, disaggregated and used for *ex vivo* mammosphere assays or intradermal re-implantation into secondary (untreated) female SCID mice at 5 × 10^5^ cell concentrations per mouse (control, *n* = 16; tamoxifen only, *n* = 15; ALM201, *n* = 7; tamoxifen plus ALM201, *n* = 6). The primary experimental outcome, i.e. time taken for tumour initiation, was recorded. The secondary experimental outcome was the number of mammospheres formed from  tumours *ex vivo* from each group. One-way ANOVA with post-hoc Dunnett’s multiple comparisons statistical test was used to compare tumour initiation and mammosphere content between control and the three treatment groups.

### Statistical analysis

Data presented are a mean of at least 3 independent experiments ± SEM. Primary sample data are from one patient; statistics were performed on 3–6 replicates. One-way ANOVA or t-test were used to assess differences between control and treatment groups. For multiple comparisons post-hoc Dunnett’s multiple comparison test was used. Statistical significance was determined by the *P* values less or equal to 0.05; *, *P* < 0.05; **, *P* < 0.01; ***, *P* < 0.001.

## Results

### FKBPL and its therapeutic peptides target CSCs and downregulate DLL4 and Notch4 in MDA-MB-231 and MCF-7 cells

**Fig. 1:**
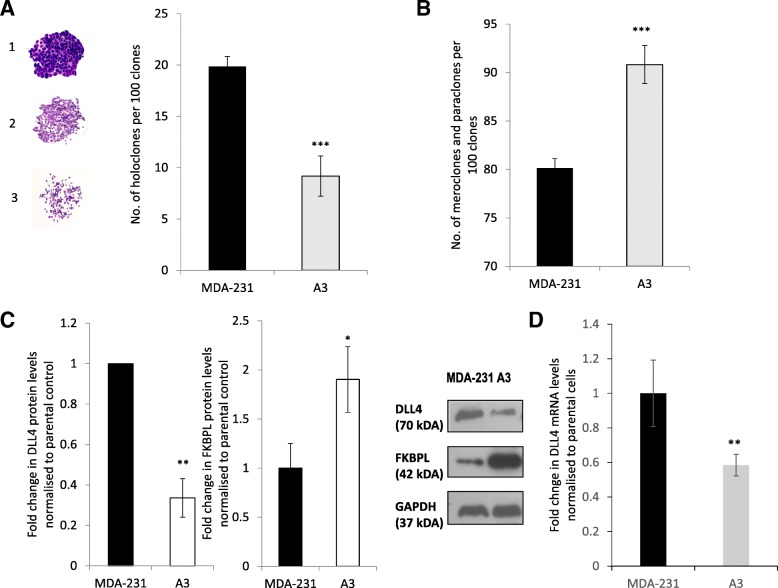
FKBPL overexpression induces differentiation of CSC-like colonies and downregulates DLL4 expression in MDA-MB-231 cells. A reduction in the number of holoclones (**a**) and a concomitant increase in the number of meroclones and paraclones (**b**) was observed, using MDA-MB-231 cells with stable FKBPL overexpression (A3), vs. MDA-MB-231 controls. 50 or 100 cells/cm^2^ per well were seeded in a six-well plate containing DMEM + 10% FCS medium and incubated for 10 days at 37°C in a humidified atmosphere of 95% O_2_/5% CO_2_ before colonies were counted manually. (representative images in the inset; 1 – holoclones; 2 – meroclones; 3- paraclones). **c** Western blot of cell lysates collected from A3 or MDA-231 cells were probed for DLL4, FKBPL and GAPDH (representative blot in inset). Protein expression was quantified using ImageJ, adjusted to GAPDH and normalised to control. **d** Real-time qPCR analysis of DLL4 mRNA levels in MDA-MB-231 vs. A3 cells. The difference in gene expression was presented as a fold change relative to the expression of the housekeeping genes, GAPDH and ß –Actin. Data points are mean ± SEM. *n* ≥ 3. * *p* < 0.05, ** *p* < 0.01, ***p < 0.01 (t-test)

**Fig. 2:**
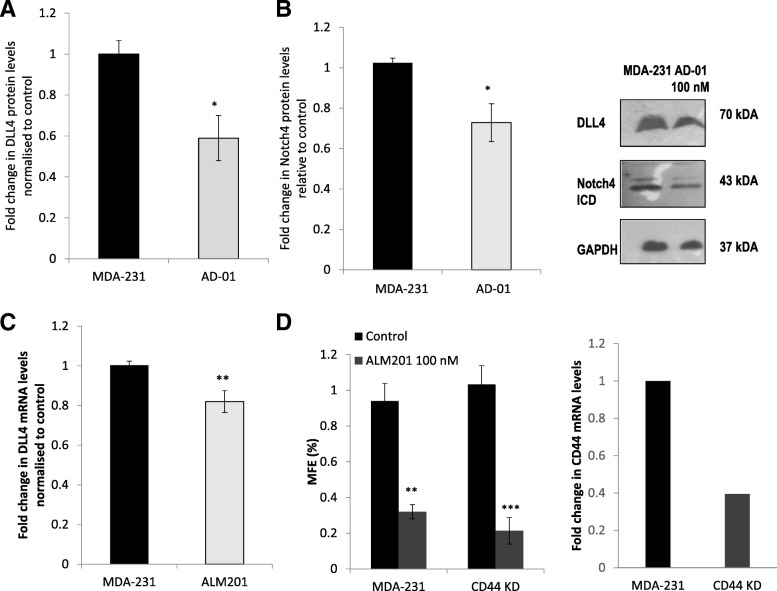
Treatment with AD-01 or ALM201 downregulates DLL4 and/or Notch 4 expression. The anti-CSC effect of ALM201 appears independent of CD44. Western blot of cell lysate from MDA-MB-231 cells treated with AD-01 (100 nM) and probed for DLL4 (**a**), Notch4ICD (**b**), and GAPDH (representative blot in inset; Notch4ICD as double band). Relative protein expression was quantified using ImageJ (t-test). **c** Real-time qPCR analysis of DLL4 mRNA levels in MDA-MB-231 cells treated with ALM201 (100 nM). The difference in gene expression was presented as a fold change relative to the expression of the housekeeping genes, GAPDH and ß –Actin (t-test). **d** Mammosphere formation in stable CD44 knockdown (KD) MDA-MB-231 cells treated with ALM201 (100 nM) vs. parental (MDA-MB-231) cells (one-way ANOVA with post-hoc Dunnett’s multiple comparisons test). Real-time qPCR analysis of CD44 mRNA levels in stable CD44 KD MDA-MB-231 cells vs. parental control; *n* = 1. Data points are mean ± SEM. *n* ≥ 3. * *p* < 0.05, ** *p* < 0.01, ***p < 0.01. ICD – intracellular domain

We have already demonstrated that FKBPL and its peptide derivatives potentially exert their activity by targeting the CD44 pathway [5, 24]. Nevertheless, when we treated MDA-MB-231 cells with a gamma-secretase inhibitor which inhibits the Notch pathway in combination with AD-01, an additive inhibitory effect on the CSCs was observed [5]. Therefore, we investigated the impact of AD-01, as well as endogenous FKBPL, using cells stably overexpressing FKBPL (A3), on DLL4 and Notch 4 levels, which are implicated in metastasis and CSC fate [11, 14]. When we stably overexpress FKBPL in MDA-MB-231 cells, the number of holoclones, which represent CD44+ CSCs [32], were reduced by over 50% (Fig. 1a, *p* < 0.001, picture 1 inset), whilst the number of meroclones and paraclones, representing differentiated cells [5], concomitantly increased (Fig. 1b, *p* < 0.001; picture 2 and 3 inset). Overall the number of colonies was unaffected. FKBPL stable overexpression in MDA-MB-231 cells also led to down-regulation of DLL4 protein (Fig. 1c, *p* < 0.01) and mRNA levels (Fig. 1d, *p* < 0.01). Similarly, treatment of MDA-MB-231 cells with AD-01 (100 nM), demonstrated inhibitory effects on both DLL4 protein (Fig. 2a, *p* < 0.05) and Notch 4 intracellular domain (ICD) protein expression (Fig. 2b, *p* < 0.05). Treatment with the clinical peptide, ALM201 (100 nM), also led to downregulation of DLL4 mRNA levels (Fig. 2c, *p* < 0.01). To elucidate whether the FKBPL-mediated effect on mammosphere forming efficiency (MFE) was dependent on CD44, ALM201 was used to treat MDA-MB-231 mammospheres with stable CD44 knockdown. To demonstrate that MDA-MB-231 mammospheres were representative of CSCs, a 10-fold enrichment in the ESA+/CD44+/CD24- subpopulation was observed using flow cytometry within MDA-MB-231 mammospheres (Additional file 1: Figure S1). ALM201 was still able to inhibit the MFE in MDA-MB-231 CD44 knockdown cells at a similar level to parental MDA-MB-231 cells (Fig. 2d, *p* < 0.01). No difference was observed between ALM201 treated cells with stable CD44 knockdown versus ALM201 treated parental cells (MDA-MB-231; Fig. 2d). This data suggests that ALM201 is not completely dependent on CD44 for its anti-CSC activity, implicating the involvement of DLL4 and Notch4, as demonstrated above.

**Fig. 3:**
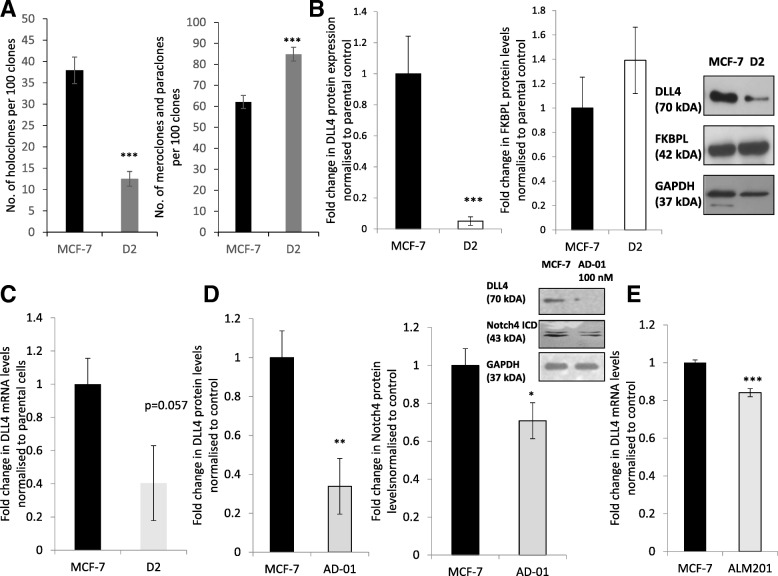
FKBPL overexpression induces differentiation of stem cell-like holoclones to more differentiated meroclones/paraclones in MCF-7. FKBPL overexpression or AD-01/ALM201 treatment downregulates DLL4 and Notch4 ICD expression in MCF-7 cells. **a** A reduction in the number of holoclones formed and a concomitant increase in the number of more differentiated, meroclone and paraclone colonies, following stable FKBPL overexpression (D2), was observed in MCF-7. **b** Western blot of cell lysates from MDA-MB-231 cells stably overexpressing FKBPL (A3) probed for DLL4, FKBPL and GAPDH (representative blot in inset). **c** Real-time qPCR analysis of DLL4 mRNA levels in MCF-7 vs. D2 cells. **d** Western blot of cell lysate from MCF-7 cells treated with AD-01 (100 nM) and probed for DLL4, Notch4ICD (double band) and GAPDH (representative blot in inset). Relative protein expression was quantified using ImageJ. **e** Real-time qPCR analysis of DLL4 mRNA levels in MCF-7 cells treated with ALM201 (100 nM). The difference in gene expression was presented as a fold change relative to the expression of the housekeeping genes, GAPDH and ß –Actin. Data points are mean ± SEM. *n* ≥ 3. * *p* < 0.05, ** *p* < 0.01, ***p < 0.01 (t-test). ICD – intracellular domain

**Fig. 4:**
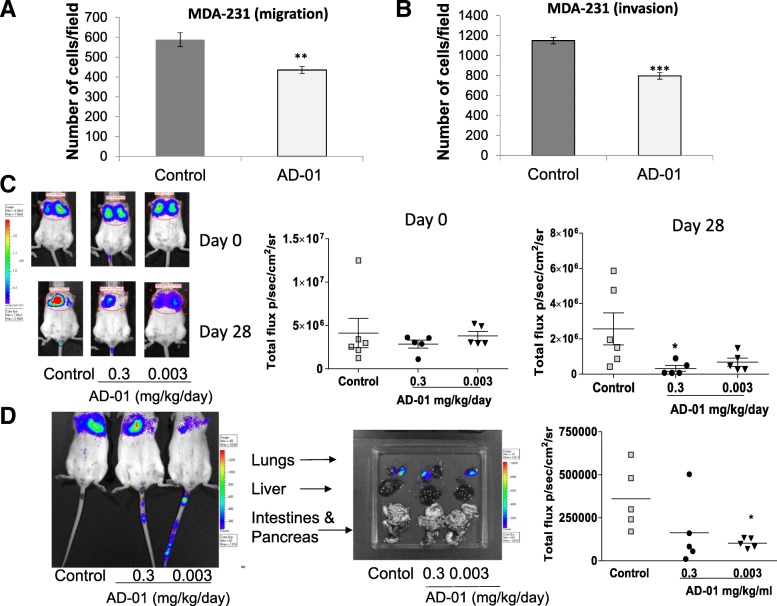
AD-01 inhibits MDA-MB-231 cell migration and invasion, and metastatic load in MDA-MB-231 mouse model of lung metastasis in SCID mice. MDA-MB-231 cell migration (**a**) and invasion (**b**) were assessed following 24 h treatment with AD-01 (1 nM) using a Boyden chamber assay. Data points are mean ± SEM. *n* ≥ 3. ** *p* < 0.01, ***p < 0.01 (t-test). **c** SCID mice were treated for 7 days with AD-01 or PBS before MDA-MB-231-LucD3H1 cells were injected i.v. Control group received PBS i.p. daily (*n* = 6) whereas treatment groups received AD-01 i.p. 0.3 mg/kg/day (*n* = 5) or 0.003 mg/kg/day (*n* = 5) for further 28 days. Lung metastasis colonization was assessed using non-invasive bioluminescence of total photon flux at day 0 and 28. A representative bioluminescent image of lungs from each group is shown inset. **d** Lung metastasis colonization was assessed using non-invasive bioluminescence of total photon flux at day 26 following inoculation of SCID mice with one million MDA-MB-231-LucD3H1 pre-treated with PBS or AD-01 (1 nM) for 1 day in vitro. Following inoculation and detection of lung metastatic load, AD-01 (0.3, *n* = 5 or 0.003 mg/kg/day; *n* = 5) or PBS (100 μl; *n* = 5) were administered i.p. daily for 26 days. Each mouse is plotted on the graph. Representative bioluminescent images of lung metastatic load and other organs from each group are shown inset. * *p* < 0.05 (one-way ANOVA with post-hoc Dunnett’s multiple comparisons test)

In ER+ breast cancer, we have shown that FKBPL is in a HSP90-associated chaperone complex with ERα receptor and that it can regulate ER signalling [22]. Using the ER+ MCF-7 cell line, FKBPL overexpression led to a better response to endocrine therapy i.e. tamoxifen and fulvestrant, whereas FKBPL knockdown had the opposite effect [22]. Endocrine therapy resistance is associated with an increase in the number of CSCs through activation of the Notch pathway [13, 33]. Here, we expand on the role of FKBPL in ER+ breast cancer by investigating the effect of stable FKBPL overexpression in MCF-7 cells (D2) on CSC-like colonies, holoclones. Similar to the triple negative breast cancer cell line, MDA-MB-231, FKBPL overexpression in MCF-7 cells resulted in a reduction in the number of holoclones and concomitant increase in the number of differentiated colonies while the overall colony number remained the same (Fig. 3a, *p* < 0.001). The effect of FKBPL stable overexpression in MCF-7 cells on DLL4 was dramatic, showing over 90% reduction in DLL4 protein expression (Fig. 3b, *p* < 0.001) and a trend towards a reduction in DLL4 mRNA levels (Fig. 3c, *p* = 0.057). In support of this, treatment of MCF-7 cells with FKBPL’s peptide derivative, AD-01, also led to downregulation of both DLL4 (*p* < 0.01) and the Notch4 ICD (*p* < 0.05) proteins levels (Fig. 3d). Similarly, the clinical peptide, ALM201, also showed downregulation of DLL4 mRNA levels in MCF-7 cells (Fig. 4e, *p* < 0.001).

### AD-01 inhibits migration, invasion and lung metastasis in triple negative MDA-MB-231 experimental models

Based on our previously published work we have established that the FKBPL pre-clinical peptide, AD-01, could inhibit both endothelial and tumour cell migration in a CD44-dependent manner [24, 25]. Furthermore, we have demonstrated that AD-01 could target CSCs in the triple negative MDA-MB-231 cell line [5]. Since CSCs are known to be associated with invasion and metastasis, here we addressed whether the FKBPL-peptide could inhibit invasion in vitro and whether this could be translated in an experimental model of metastasis using the triple negative metastatic MDA-MB-231 breast cancer cells. Treatment of MDA-MB-231 cells for 24 h with AD-01 (1 nM) confirmed inhibition of cell migration, the first step in the invasive process, through an uncoated Boyden chamber (Fig. 4a, *n* ≥ 3, *p* < 0.01). Furthermore, we were also able to prevent invasion through a Matrigel coated Boyden chamber (Fig. 4b, *n* ≥ 3, *p* < 0.001). Since it has been previously demonstrated by Ebos and colleagues [34] that anti-angiogenic agents can promote metastases, we investigated whether AD-01 pre-treatment can prevent metastatic invasion using two separate in vivo MDA-MB-231 experimental lung metastasis models. In the first experiment, SCID mice were pre-treated daily with the stated dose of AD-01 or PBS for 1 week prior to being inoculated with MDA-MB-231-lucD3H1 cells via tail vein injection. AD-01 was subsequently administered to mice daily via i.p. injection (0.03 and 0.3 mg/kg/day). Control mice received PBS injections daily. AD-01 (0.3 mg/kg/day, *p* < 0.05; 0.003 mg/kg/day, *p* = 0.08) inhibited lung colonization of breast cancer cells following 28 days of in vivo treatment in addition to pre-treatment in vitro (Fig. 4c). No difference in the total photon flux was observed at day 0 following intravenous inoculation of MDA-231-lucD3H1 cells from either of the pre-treated groups (PBS or AD-01; Fig. 4c). In the second experiment, MDA-MB-231-lucD3H1 cells were pre-treated for 24 h with the stated dose of AD-01 or PBS, and injected via tail vein (in this experiment the mice were not pre-treated). Following i.v. injection of the cells, mice were treated i.p. in vivo for 26 days using either PBS as a control or AD-01 (0.3 or 0.003 mg/kg/day). Lung metastasis colonization was assessed using non-invasive bioluminescence of total photon flux. AD-01 (0.003 mg/kg/day; *p* < 0.05) treatment significantly reduced the total photon flux, indicative of the lung cell load compared to the vehicle PBS control (Fig. 4d, *p* < 0.05). Weight and wellbeing of each mouse was monitored daily and no significant weight reduction (≥15%) was observed. The drug was generally well tolerated and all animals where initial metastatic burden was recorded following MDA-MB-231-lucD3H1 cell inoculation via tail vein were included in the analysis.

### FKBPL and its therapeutic peptides target endocrine therapy-resistant CSCs within an ER+ breast cancer context in both cell lines and clinical samples

**Fig. 5:**
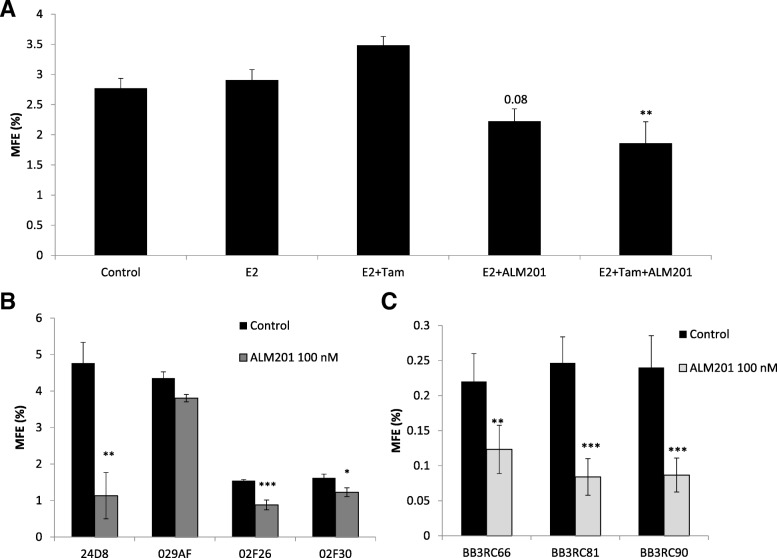
Tamoxifen shows no effect on the number of mammospheres formed whereas ALM201 alone or in combination with tamoxifen reduces the number of mammosphere in MCF-7 cells and patient samples. **a** MCF-7 cells were treated with estradiol (100 nM; E2) ± tamoxifen (1 μM; Tam) ± ALM201 (1 nM) in a monolayer for 72 h before the cells were used in the mammosphere assay. Mammospheres formed were counted manually 3–5 days later (one-way ANOVA with post-hoc Dunnett’s multiple comparisons test). **b** Mammospheres formed from cancer cells derived from individual primary breast cancer sample treated neoadjuvantly with letrozole were counted in vitro following treatment with ALM201 (100 nM) or control for 5–7 days; *n* ≥ 5 replicates (t-test). **c** Mammospheres formed from cancer cells derived from pleural effusions from individual breast cancer patients (ER+ metastatic breast cancer samples) were counted following treatment with ALM201 (100 nM) or control for 7 days; *n* ≥ 5 replicates (t-test). Data points are mean ± SEM. *n* ≥ 3. * *p* < 0.05, ** *p* < 0.01, *** *p* < 0.001

CSCs within ER+ breast cancer are resistant to endocrine therapy due to the lack of ER expression [35]. In order to demonstrate that FKBPL-based clinical peptide, ALM201, is able to target endocrine therapy resistant CSCs in ER+ breast cancer, we treated the ER+ breast cancer cell line, MCF-7, with estradiol (100 nM) ± tamoxifen (1 μM) ± ALM201 (1 nM) and carried out mammosphere assay. A trend towards increase in the MFE was demonstrated following treatment with tamoxifen alone (MFE = 3.5%, *p* = 0.17) in the presence of estradiol (Fig. 5a). ALM201 alone, at a very low dose (1 nM) (MFE = 2.2%, *p* = 0.08) or in combination with tamoxifen (MFE = 1.86%, *p* < 0.01) reduced the MFE compared to estradiol treatment alone (Fig. 5a); the statistical significance was only observed when tamoxifen and ALM201 were used in combination. Importantly, the combination of ALM201 and tamoxifen seems to be even more effective at inhibiting the MFE (%). To ensure that mammospheres were representative of the CSC population, we were able to demonstrate a two-fold enrichment in the CD44^+^/CD24^−^ subpopulation of cells within MCF-7 mammospheres (Additional file 2: Figure S2).

Previously, using a range of ER+ and ER- metastatic breast cancer patient samples we demonstrated a modest 20% reduction in the MFE following AD-01 treatment, even though the dose of AD-01 used was low (5 nM) [5]. Here we assessed the effects of ALM201, at a dose of 100 nM using clinically relevant ER+ breast cancer tissue from patients undergoing mastectomy and treated in the neoadjuvant setting with letrozole. We demonstrated up to 12-fold higher MFE (MFE ranged from 1.5–4.8; Fig. 5b- black bars) compared to our previously published data using breast cancer tissue from patients without neoadjuvant treatment (where MFEs of 0.4 were observed) [5]. Importantly, in three of the four patient samples, ALM201 significantly reduced the MFE, up to 70% (Fig. 5b). Sample 029AF, where no significant effect was observed, was negative for the expression of PR unlike the rest of the samples which were all ER+ and PR+ (Additional file 3: Table S1).

The anti-CSC effect of ALM201 was further validated in ER+ metastatic breast cancer samples from pleural effusions. ALM201 (100 nM) was effective at reducing the MFE by over 45% in three patient samples (BB3EC66–45% reduction; BB3RC8–66% reduction; BB3RC90–64% reduction; *p* < 0.01 or 0.001; Fig. 5c). This is important, since these samples are from patients with end-stage, highly metastatic disease with treatment-resistant tumours; all of these patients were unsuccessfully treated with a wide range of endocrine and chemotherapy regimens (Additional file 4: Table S2).

### ALM201 in combination with tamoxifen delays tumour initiation and reduces the number of mammosphere forming tamoxifen-resistant CSCs in ER+ MCF-7 xenografts

**Fig. 6:**
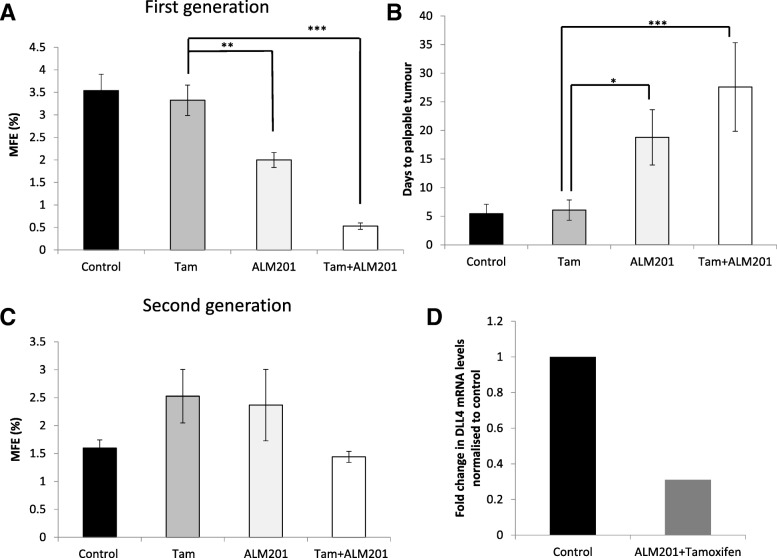
ALM201 alone or in combination with tamoxifen delays tumour recurrence in vivo which correlates with reduced number of mammospheres *ex vivo*. **a** Established MCF-7 xenografts (100–150 mm^3^) were treated with vehicle control (*n* = 5), tamoxifen (*n* = 4) or ALM201 (*n* = 4) alone or in combination (*n* = 4) for 21 days. Mammosphere formation was assessed *ex vivo* following excision and disaggregation of established MCF-7 xenografts; *n* ≥ 3 replicates per mouse. **b** Tumour cells from the treated xenografts were re-implanted into secondary mice and tumour occurrence was monitored twice a week and time to tumour initiation calculated (vehicle control, *n* = 13; tamoxifen (Tam), *n* = 14; ALM201, *n* = 5; tamoxifen + ALM201 (Tam + ALM201), *n* = 5). **c** Mammosphere formation following excision and disaggregation of established MCF-7 xenografts from the second generation mice without any further treatment in vivo (control, *n* = 6; Tam, *n* = 4; ALM201, *n* = 2; Tam + ALM201, *n* = 3); *n* ≥ 3 replicates per mouse. **d** Real-time qPCR analysis of DLL4 in MCF-7 xenografts treated with tamoxifen and ALM201 in vivo (*n* = 2). The difference in gene expression was presented as a fold change relative to the expression of the housekeeping genes, GAPDH and ß -Actin. Data points are mean ± SEM. *n* ≥ 3. * *p* < 0.05, ** *p* < 0.01, *** *p* < 0.001 (one-way ANOVA with post-hoc Dunnett’s multiple comparisons test)

In order to validate the results obtained in vitro and in clinical samples, an in vivo tumour initiation assay was carried out. Here, mice carrying established tumours (100–150 mm^3^) were treated with 1) vehicle control, 2) tamoxifen (12.5 mg/kg/day), 3) ALM201 (0.3 mg/kg/day), and 4) tamoxifen + ALM201, for a period of 21 days. Following three weeks of treatment, tumours were excised and tumour cells used in an *ex vivo* mammosphere assay or re-implanted into second generation SCID mice without any further treatment to assess the tumour initiating potential. The *ex vivo* mammosphere assay, using tumour cells from first generation treated MCF-7 xenografts, showed no change in the MFE between control and tamoxifen treated tumours (MFE = 3.5%, control (*n* = 6) vs. MFE =3.3%, tamoxifen (*n* = 4); Fig. 6a). ALM201 alone or in combination with tamoxifen led to a significant reduction in the MFE (MFE = 2%, ALM201 (*n* = 4), *p* < 0.01; and MFE = 0.5%, ALM201 and tamoxifen (*n* = 4), *p* < 0.001; Fig. 6a) compared to tamoxifen treatment. Interestingly, the combination of tamoxifen and ALM201 appeared even more effective at inhibiting the MFE than ALM201 alone (Fig. 6a, *p* < 0.01). When tumour cells were re-implanted into the second generation untreated mice, there was no delay in the number of days to palpable tumours between vehicle-treated or tamoxifen-treated tumour cells (Fig. 6b), suggesting that tamoxifen does not target the tumour initiating cell population. However, cells derived from the first generation ALM201-treated mice showed a significant delay in tumour recurrence of ~ 12 days compared to control or tamoxifen (Fig. 6b, *p* < 0.05). Importantly, the time to palpable tumour was even further delayed by 22 days when cells from the first generation tamoxifen and ALM201 treated mice were used in combination compared to tamoxifen alone (Fig. 6b, *p* < 0.001). Weight and wellbeing of each mouse were monitored closely and no significant weight reductions (≥15%) were observed. Any mouse showing signs of poor wellbeing was euthanized according to the approved protocol. The drugs was generally well tolerated and all animals displaying tumours were included in the analysis; vehicle-treated/control (13/16), tamoxifen (14/15), ALM201 (5/7) and tamoxifen+ALM201 (5/6).

When secondary tumours were excised and tumour cells were subjected to an *ex vivo* mammosphere assay, no effect on the MFE was observed in the tamoxifen-treated group (*n* = 4, *p* = 0.1) compared to control (*n* = 6; Fig. 6c). However, in combination with ALM201, the MFE appeared reduced compared to tamoxifen alone (*n* = 3; Fig. 6c, *p* = 0.15), however not statistically significant. Treatment with ALM201 alone did not lead to any significant change in the MFE compared to control (*n* = 2; Fig. 6c). This could be due to the small number of tumours excised or interrupted treatment with ALM201 in the second generation mice. Interestingly, *ex vivo* qPCR analysis of MCF-7 xenografts treated with both ALM201 and tamoxifen also showed a trend towards downregulation of DLL4 mRNA compared to control (Fig. 6d; *n* = 2).

## Discussion

We have previously demonstrated a role for FKBPL in ER signalling, endocrine therapy response, angiogenesis and CSC differentiation [5, 22, 24]. To date, the mechanism of action has been attributed to a potential role in the CD44 pathway and stabilisation of p21 [5, 22, 36]. In addition to this, we have shown that high FKBPL levels are associated with a positive prognosis in breast cancer [21]. In this study, for the first time, we assessed the pre-clinical activity of novel systemic anti-cancer therapeutic peptides, ALM201 & AD-01, in the metastatic setting, and highlighted their impact on endocrine therapy resistant cancer stem cells; both areas of unmet clinical need. These effects were demonstrated using a range of experiments with cell lines, primary breast cancer samples and in vivo models.

In triple negative breast cancer using MDA-MB-231 cells, we demonstrated FKBPL-mediated differentiation of CSCs to more “mature” cancer cells, no cytotoxic effect and inhibition of cell migration, invasion and metastasis. In our in vivo lung metastasis model we demonstrated that pre-treatment with AD-01 prevents lung colonization of breast cancer cells which is, likely, through prevention of engraftment of the tumour cells given AD-01’s inhibitory effect on cell migration and invasion. In ER+ breast cancer, using MCF-7 cells and ER+ breast cancer samples, we also demonstrated FKBPL-mediated CSC differentiation, inhibition of CSCs resistant to endocrine therapy and delay in tumour initiation. Interestingly, ALM201 in combination with tamoxifen appeared even more effective at inhibiting CSC population than ALM201 alone while tamoxifen shows no effect on CSCs. Furthermore, FKBPL appears to downregulate DLL4 and Notch 4 levels, which has not been previously reported. Therefore, we identified a novel role for FKBPL in reducing the metastatic burden which could be linked to the inhibition of CSCs and the regulation of CD44, DLL4 and Notch 4. This is very important since other anti-angiogenic agents show increased metastatic potential [34]. CSCs have been implicated in cancer metastases, as the primary cells likely to migrate and populate metastatic sites, due to their strong migratory and pluripotent potential [37]. High Notch activity has been implicated in cancer pathogenesis and Notch 4 is specifically active within breast CSCs [11, 38]. Moreover, both Notch and CD44 have been implicated in hypoxia-driven enrichment of CSC population, tumour recurrence and enhanced metastatic phenotype after treatment with anti-angiogenic agents or hypoxia inducible factors [39–41]. Our data suggests that FKBPL-based peptides in addition to their well-established anti-angiogenic [24, 25] and anti-CSC activity [5] via CD44, are able to inhibit lung metastasis, possibly by modulating the Notch pathway members, DLL4 and Notch 4, within breast cancer, giving these agents a potential competitive advantage. Further studies would be required to elucidate the role of FKBPL/ALM201/AD-01 in Notch 4 and DLL4 signalling.

Furthermore, our in vivo data in relation to tamoxifen treatment confirms that tamoxifen does not target CSCs or inhibit tumour initiation. Conversely, ALM201 alone or in combination with tamoxifen demonstrated a substantial delay in tumour initiation and reduced the proportion of the CSC-like population assessed by *ex vivo* mammosphere assay, which correlates with the content of CD44^+^/CD24^−^ CSC population. The combination of tamoxifen and ALM201 had a more pronounced inhibitory effect on tumour initiation and the CSC-like population compared to ALM201 alone, thus suggesting that this combination might be advantageous clinically. Notch inhibitors have already demonstrated activity in combination with tamoxifen, and Notch 4, in particular, has been implicated as a viable target to prevent metastasis in tamoxifen-resistance breast cancer [42, 43]. Nevertheless, correlation between the activity of Notch ligands, receptors and target genes is complex and elucidating the functional role for individual Notch receptors and ligands in mediating resistance to therapy, tumour recurrence or metastasis in breast cancer is necessary [44, 45]. Our data suggests that FKBPL can specifically downregulate DLL4 and intracellular Notch 4, however the effects on other important members of the Notch pathways and Notch signalling needs to be investigated further.

In summary, based on the results obtained in this study and previously published studies, while the novel FKBPL-based anti-cancer therapeutic peptides, ALM201 and AD-01, are not cytotoxic, these agents have multiple synergistic anti-tumour activities including anti-angiogenic, anti-CSC and anti-metastatic involving CD44, and possibly, DLL4 and Notch 4 which gives them a clinical advantage over other anti-angiogenic agents.

## Conclusions

FKBPL-derived therapeutic peptides, AD-01/ALM201, demonstrate significant anti-angiogenic, anti-CSC activity and, now, anti-metastatic activity and therefore have enhanced clinical utility in comparison to clinically available anti-angiogenic agents. This triple therapeutic action will undoubtedly provide added clinical benefit as it progresses through clinical development. Based on robust pre-clinical efficacy, without associated toxicity, ALM201 entered a ‘first in man’ clinical trial in oncology, where unlike other anti-angiogenics, it is not cytotoxic and displayed an excellent safety profile in this Phase I clinical trial [27].

## Additional files

Additional file 1: **Figure S1.** The content of ESA^+^/CD44^+^/CD24^−^ cell population within mammosphere culture in MDA-MB-231 cells. (JPG 78 kb)

Additional file 2: **Figure S2.** The content of CD44^+^/CD24^−^ cell population within mammosphere culture in MCF-7 cells. (JPG 89 kb)

Additional file 3: **Table S1.** The clinical characteristic of primary samples used in the study. (JPG 153 kb)

Additional file 4: **Table S2.** The treatment regimens used in metastatic ER+ patient samples. (JPG 97 kb)

## Abbreviations

BCSS: Breast cancer specific survival
BSA: Bovine serum albumin
CSCs: Cancer stem cells
DMEM: Dulbecco’s modified Eagle’s medium
E: Estradiol
EGF: Epidermal growth factor
EGFR: Epidermal growth factor receptor
ER: Estrogen receptor alpha
ESA: Epithelial specific antigen
ICD: Intracellular domain
MFE: Mammosphere forming efficiency
PBS: Phosphate-buffered solution
T: Tamoxifen

## References

[1] Dai X, Li T, Bai Z, Yang Y, Liu X, Zhan J, et al. Breast cancer intrinsic subtype classification, clinical use and future trends. Am J Cancer Res [Internet]. e-Century Publishing Corporation; 2015 [cited 2018 Jul 8];5(10):2929–43. Available from: http://www.ncbi.nlm.nih.gov/pubmed/26693050. PMC4656721 26693050

[2] Curtis C, Shah SP, Chin S-F, Turashvili G, Rueda OM, Dunning MJ, et al. The genomic and transcriptomic architecture of 2,000 breast tumours reveals novel subgroups. Nature [Internet]. Nature Publishing Group; 2012;486(7403):346. Available from: 10.1038/nature10983 10.1038/nature10983 PMC3440846 22522925

[3] Reya T, Morrison SJ, Clarke MF, Weissman IL. Stem cells, cancer, and cancer stem cells. Nature [Internet]. 2001 Nov 1 [cited 2018 Apr 12];414(6859):105–111. Available from: http://www.ncbi.nlm.nih.gov/pubmed/11689955. 10.1038/35102167 11689955

[4] Zeichner SB, Terawaki H, Gogineni K. A Review of Systemic Treatment in Metastatic Triple-Negative Breast Cancer. Breast Cancer (Auckl) [Internet]. SAGE Publications; 2016 [cited 2018 Jul 8];10:25–36. Available from: http://www.ncbi.nlm.nih.gov/pubmed/27042088. 10.4137/BCBCR.S32783 PMC4807882 27042088

[5] McClements L, Yakkundi A, Papaspyropoulos A, Harrison H, Ablett MP, Jithesh P V, et al. Targeting treatment-resistant breast cancer stem cells with FKBPL and its peptide derivative, AD-01, via the CD44 pathway. Clin Cancer Res [Internet]. 2013 Jul 15 [cited 2014 Nov 12];19(14):3881–93. Available from: http://www.ncbi.nlm.nih.gov/pubmed/23741069. 10.1158/1078-0432.CCR-13-0595 23741069

[6] Oh E, Kim Y-J, An H, Sung D, Cho T-M, Farrand L, et al. Flubendazole elicits anti-metastatic effects in triple-negative breast cancer via STAT3 inhibition. Int J Cancer [Internet]. 2018 May 9 [cited 2018 Jul 8]; Available from: http://www.ncbi.nlm.nih.gov/pubmed/29744876. 10.1002/ijc.31585 29744876

[7] Alferez DG, Simões BM, Howell SJ, Clarke RB. The Role of Steroid Hormones in Breast and Effects on Cancer Stem Cells. Curr Stem Cell Reports [Internet]. 2018 [cited 2018 Apr 12];4(1):81–94. Available from: http://www.ncbi.nlm.nih.gov/pubmed/29600163. 10.1007/s40778-018-0114-z PMC5866269 29600163

[8] Hiscox S, Jiang WG, Obermeier K, Taylor K, Morgan L, Burmi R, et al. Tamoxifen resistance in MCF7 cells promotes EMT-like behaviour and involves modulation of beta-catenin phosphorylation. Int J cancer [Internet]. 2006 [cited 2018 Apr 12];118(2):290–301. Available from: http://doi.wiley.com/10.1002/ijc.21355 10.1002/ijc.21355 16080193

[9] Creighton CJ, Li X, Landis M, Dixon JM, Neumeister VM, Sjolund A, et al. Residual breast cancers after conventional therapy display mesenchymal as well as tumor-initiating features. Proc Natl Acad Sci [Internet]. 2009 18 [cited 2018 Apr 12];106(33):13820–5. Available from: http://www.pnas.org/cgi/doi/10.1073/pnas.0905718106 10.1073/pnas.0905718106 PMC2720409 19666588

[10] Acar A, Simões BM, Clarke RB, Brennan K, Brennan K. A Role for Notch Signalling in Breast Cancer and Endocrine Resistance. Stem Cells Int [Internet]. Hindawi Publishing Corporation; 2016 [cited 2017 Jan 9];2016:1–6. Available from: http://www.hindawi.com/journals/sci/2016/2498764/10.1155/2016/2498764 PMC4736972 26880941

[11] Harrison H, Farnie G, Howell SJ, Rock RE, Stylianou S, Brennan KR, et al. Regulation of breast cancer stem cell activity by signaling through the Notch4 receptor. Cancer Res [Internet]. Europe PMC Funders; 2010 [cited 2018 may 4];70(2):709–18. Available from: http://www.ncbi.nlm.nih.gov/pubmed/20068161. 10.1158/0008-5472.CAN-09-1681 PMC3442245 20068161

[12] Lombardo Y, Faronato M, Filipovic A, Vircillo V, Magnani L, Coombes RC. Nicastrin and Notch4 drive endocrine therapy resistance and epithelial to mesenchymal transition in MCF7 breast cancer cells. Breast Cancer Res [Internet]. 2014 [cited 2018 Apr 20];16(3):R62. Available from: http://www.ncbi.nlm.nih.gov/pubmed/24919951. 10.1186/bcr3675 PMC4095694 24919951

[13] Simões BM, O’Brien CS, Eyre R, Silva A, Yu L, Sarmiento-Castro A, et al. Anti-estrogen Resistance in Human Breast Tumors Is Driven by JAG1-NOTCH4-Dependent Cancer Stem Cell Activity. Cell Rep [Internet]. 2015 Sep [cited 2016 Nov 2];12(12):1968–77. Available from: http://linkinghub.elsevier.com/retrieve/pii/S221112471500947X 10.1016/j.celrep.2015.08.050 PMC4594158 26387946

[14] Xiao M, Yang S, Ning X, Huang Y. Aberrant expression of δ-like ligand 4 contributes significantly to axillary lymph node metastasis and predicts postoperative outcome in breast cancer. Hum Pathol [Internet]. 2014 [cited 2018 Apr 13];45(11):2302–10. Available from: http://www.ncbi.nlm.nih.gov/pubmed/25260720. 10.1016/j.humpath.2014.04.025 25260720

[15] Kontomanolis E, Panteliadou M, Giatromanolaki A, Pouliliou S, Efremidou E, Limberis V, et al. Delta-like ligand 4 (DLL4) in the plasma and neoplastic tissues from breast cancer patients: correlation with metastasis. Med Oncol [Internet]. 2014 [cited 2018 Apr 20];31(5):945. Available from: http://www.ncbi.nlm.nih.gov/pubmed/24696220. 10.1007/s12032-014-0945-0 24696220

[16] Wang Q, Shi Y, Butler H, Xue J, Wang G, Duan P, et al. Role of delta-like ligand-4 in chemoresistance against docetaxel in MCF-7 cells. Hum Exp Toxicol [Internet]. 2017 [cited 2018 Apr 20];36(4):328–38. Available from: http://www.ncbi.nlm.nih.gov/pubmed/27334972. 10.1177/0960327116650006 27334972

[17] Xu Z, Wang Z, Jia X, Wang L, Chen Z, Wang S, et al. MMGZ01, an anti-DLL4 monoclonal antibody, promotes nonfunctional vessels and inhibits breast tumor growth. Cancer Lett [Internet]. 2016 [cited 2018 Apr 20];372(1):118–127. Available from: http://www.ncbi.nlm.nih.gov/pubmed/26739060. 10.1016/j.canlet.2015.12.025 26739060

[18] Miao Z-F, Xu H, Xu H-M, Wang Z-N, Zhao T-T, Song Y-X, et al. DLL4 overexpression increases gastric cancer stem/progenitor cell self-renewal ability and correlates with poor clinical outcome via Notch-1 signaling pathway activation. Cancer Med [Internet]. 2017 Jan [cited 2018 Apr 12];6(1):245–57. Available from: http://doi.wiley.com/10.1002/cam4.962 10.1002/cam4.962 PMC5269703 27891816

[19] Robson T, James IF. The therapeutic and diagnostic potential of FKBPL; a novel anticancer protein. Drug Discov Today [Internet]. Elsevier Ltd; 2012 [cited 2014 Nov 12];17(11–12):544–8. Available from: http://www.ncbi.nlm.nih.gov/pubmed/22265918. 10.1016/j.drudis.2012.01.002 22265918

[20] McClements L, Annett S, Yakkundi A, Robson T. The Role of Peptidyl Prolyl Isomerases in Aging and Vascular Diseases. Curr Mol Pharmacol [Internet]. 2015 May 19 [cited 2015 May 20]; Available from: http://www.ncbi.nlm.nih.gov/pubmed/25986561. 10.2174/1874467208666150519115729 25986561

[21] Nelson L, McKeen HD, Marshall A, Mulrane L, Starczynski J, Storr SJ, et al. FKBPL: a marker of good prognosis in breast cancer. Oncotarget [Internet]. 2015 May 20 [cited 2015 Jul 1];6(14):12209–23. Available from: http://www.ncbi.nlm.nih.gov/pubmed/25906750. 10.18632/oncotarget.3528 PMC4494933 25906750

[22] McKeen HD, Byrne C, Jithesh P V, Donley C, Valentine A, Yakkundi A, et al. FKBPL regulates estrogen receptor signaling and determines response to endocrine therapy. Cancer Res [Internet]. 2010 [cited 2014 Nov 12];70(3):1090–100. Available from: http://www.ncbi.nlm.nih.gov/pubmed/20103631. 10.1158/0008-5472.CAN-09-2515 20103631

[23] Donley C, McClelland K, McKeen HD, Nelson L, Yakkundi A, Jithesh P V, et al. Identification of RBCK1 as a novel regulator of FKBPL: implications for tumor growth and response to tamoxifen. Oncogene [Internet]. Nature Publishing Group; 2014 Jun 26 [cited 2014 Nov 12];33(26):3441–50. Available from: http://www.ncbi.nlm.nih.gov/pubmed/23912458. 10.1038/onc.2013.306 23912458

[24] Valentine A, O’Rourke M, Yakkundi A, Worthington J, Hookham M, Bicknell R, et al. FKBPL and peptide derivatives: novel biological agents that inhibit angiogenesis by a CD44-dependent mechanism. Clin Cancer Res [Internet]. 2011 [cited 2014 Nov 12];17(5):1044–56. Available from: http://www.pubmedcentral.nih.gov/articlerender.fcgi?artid=3059488&tool=pmcentrez&rendertype=abstract 10.1158/1078-0432.CCR-10-2241 PMC3059488 21364036

[25] Yakkundi A, McCallum L, O’Kane A, Dyer H, Worthington J, McKeen HD, et al. The Anti-Migratory Effects of FKBPL and Its Peptide Derivative, AD-01: Regulation of CD44 and the Cytoskeletal Pathway. PLoS One. 2013;8(2). https://www.ncbi.nlm.nih.gov/pubmed/23457460. 10.1371/journal.pone.0055075 PMC3574160 23457460

[26] Li W, Ma H, Zhang J, Zhu L, Wang C, Yang Y. Unraveling the roles of CD44/CD24 and ALDH1 as cancer stem cell markers in tumorigenesis and metastasis. Sci Rep [Internet]. 2017 [cited 2018 Apr 20];7(1):13856. Available from: http://www.ncbi.nlm.nih.gov/pubmed/29062075. 10.1038/s41598-017-14364-2 PMC5653849 29062075

[27] El-Helali A, Plummer R, Jayson G, Coyle V, Drew Y, Mescallado N HN, Clamp A, McCann J, Kennedy R, Cranston A WR. A phase I dose-escalation study of the novel peptide ALM201 in patients (pts) with advanced solid tumours. In: A phase I dose-escalation study of the novel peptide ALM201 in patients (pts) with advanced solid tumours [Internet]. Developmental therapeutics; 2017. p. Supplement 5. Available from: https://academic.oup.com/annonc/article/28/suppl_5/mdx367.017/4108608.

[28] Piva M, Domenici G, Iriondo O, Rábano M, Simões BM, Comaills V, et al. Sox2 promotes tamoxifen resistance in breast cancer cells. EMBO Mol Med [Internet]. 2014 Jan [cited 2018 Apr 12];6(1):66–79. Available from: http://www.ncbi.nlm.nih.gov/pubmed/24178749. 10.1002/emmm.201303411 PMC3936493 24178749

[29] Li X, Xu Y, Chen Y, Chen S, Jia X, Sun T, et al. SOX2 promotes tumor metastasis by stimulating epithelial-to-mesenchymal transition via regulation of WNT/β-catenin signal network. Cancer Lett [Internet]. 2013 19 [cited 2018 Apr 20];336(2):379–389. Available from: http://linkinghub.elsevier.com/retrieve/pii/S0304383513002577 10.1016/j.canlet.2013.03.027 23545177

[30] Liu K, Xie F, Gao A, Zhang R, Zhang L, Xiao Z, et al. SOX2 regulates multiple malignant processes of breast cancer development through the SOX2/miR-181a-5p, miR-30e-5p/TUSC3 axis. Mol Cancer [Internet]. 2017 Dec 14 [cited 2018 Apr 20];16(1):62. Available from: http://www.ncbi.nlm.nih.gov/pubmed/28288641. 10.1186/s12943-017-0632-9 PMC5348847 28288641

[31] McFarlane S, Coulter JA, Tibbits P, O’Grady A, McFarlane C, Montgomery N, et al. CD44 increases the efficiency of distant metastasis of breast cancer. Oncotarget [Internet] 2015;6(13):11465–76. Available from: https://www.ncbi.nlm.nih.gov/pubmed/25888636. 10.18632/oncotarget.3410 PMC4484469 25888636

[32] Khan GN, Kim EJ, Shin TS, Lee SH. Heterogeneous Cell Types in Single-cell-derived Clones of MCF7 and MDA-MB-231 Cells. Anticancer Res [Internet]. 2017 [cited 2018 Apr 20];37(5):2343–2354. Available from: http://www.ncbi.nlm.nih.gov/pubmed/28476800. 10.21873/anticanres.11572 28476800

[33] O’Brien CS, Farnie G, Howell SJ, Clarke RB. Breast Cancer Stem Cells and Their Role in Resistance to Endocrine Therapy. Horm Cancer [Internet]. 2011 22 [cited 2016 Nov 2];2(2):91–103. Available from: http://link.springer.com/10.1007/s12672-011-0066-6 10.1007/s12672-011-0066-6 PMC10358078 21761332

[34] Ebos JML, Lee CR, Cruz-Munoz W, Bjarnason GA, Christensen JG, Kerbel RS. Accelerated metastasis after short-term treatment with a potent inhibitor of tumor angiogenesis. Cancer Cell [Internet]. 2009 [cited 2018 Nov 14];15(3):232–239. Available from: http://linkinghub.elsevier.com/retrieve/pii/S1535610809000294 10.1016/j.ccr.2009.01.021 PMC4540346 19249681

[35] Harrison H, Simões BM, Rogerson L, Howell SJ, Landberg G, Clarke RB. Oestrogen increases the activity of oestrogen receptor negative breast cancer stem cells through paracrine EGFR and Notch signalling. Breast Cancer Res [Internet]. 2013 [cited 2016 Nov 2];15(2):R21. Available from: http://breast-cancer-research.biomedcentral.com/articles/10.1186/bcr3396 10.1186/bcr3396 PMC3672803 23497505

[36] Yakkundi A, Bennett R, Hernández-Negrete I, Delalande J-M, Hanna M, Lyubomska O, et al. FKBPL Is a Critical Antiangiogenic Regulator of Developmental and Pathological Angiogenesis. Arterioscler Thromb Vasc Biol [Internet]. 2015 Mar 12 [cited 2015 mare 25];35(4):845–854. Available from: http://www.ncbi.nlm.nih.gov/pubmed/25767277. 10.1161/ATVBAHA.114.304539 PMC4415967 25767277

[37] Geng S-Q, Alexandrou AT, Li JJ. Breast cancer stem cells: Multiple capacities in tumor metastasis. Cancer Lett [Internet]. 2014 Jul 10 [cited 2018 Jul 9];349(1):1–7. Available from: http://www.ncbi.nlm.nih.gov/pubmed/24727284. 10.1016/j.canlet.2014.03.036 PMC4177877 24727284

[38] Harrison H, Farnie G, Brennan KR, Clarke RB. Breast Cancer Stem Cells: Something Out of Notching? Cancer Res [Internet]. 2010 [cited 2016 Nov 2];70(22):8973–8976. Available from: http://cancerres.aacrjournals.org/cgi/doi/10.1158/0008-5472.CAN-10-1559 10.1158/0008-5472.CAN-10-1559 21045140

[39] Kitajima S, Lee KL, Fujioka M, Sun W, You J, Chia GS, et al. Hypoxia-inducible factor-2 alpha up-regulates CD70 under hypoxia and enhances anchorage-independent growth and aggressiveness in cancer cells. Oncotarget [Internet]. 2018 [cited 2018 may 6];9(27):19123–19135. Available from: http://www.ncbi.nlm.nih.gov/pubmed/29721188. 10.18632/oncotarget.24919 PMC5922382 29721188

[40] Gustafsson M V, Zheng X, Pereira T, Gradin K, Jin S, Lundkvist J, et al. Hypoxia requires notch signaling to maintain the undifferentiated cell state. Dev Cell [Internet]. Elsevier; 2005 Nov 1 [cited 2018 may 6];9(5):617–28. Available from: http://www.ncbi.nlm.nih.gov/pubmed/16256737. 10.1016/j.devcel.2005.09.010 16256737

[41] Harrison H, Rogerson L, Gregson HJ, Brennan KR, Clarke RB, Landberg G. Contrasting Hypoxic Effects on Breast Cancer Stem Cell Hierarchy Is Dependent on ER- Status. Cancer Res [Internet]. 2013 [cited 2018 May 6];73(4):1420–1433. Available from: http://cancerres.aacrjournals.org/cgi/doi/10.1158/0008-5472.CAN-12-2505 10.1158/0008-5472.CAN-12-2505 23248117

[42] Haughian JM, Pinto MP, Harrell JC, Bliesner BS, Joensuu KM, Dye WW, et al. Maintenance of hormone responsiveness in luminal breast cancers by suppression of Notch. Proc Natl Acad Sci U S A [Internet]. 2012 21 [cited 2018 May 6];109(8):2742–2747. Available from: http://www.pnas.org/cgi/doi/10.1073/pnas.1106509108 10.1073/pnas.1106509108 PMC3287001 21969591

[43] Bui QT, Im JH, Jeong SB, Kim Y-M, Lim SC, Kim B, et al. Essential role of Notch4/STAT3 signaling in epithelial–mesenchymal transition of tamoxifen-resistant human breast cancer. Cancer Lett [Internet]. 2017 Apr 1 [cited 2018 may 6];390:115–125. Available from: http://www.ncbi.nlm.nih.gov/pubmed/28108315. 10.1016/j.canlet.2017.01.014 28108315

[44] Acar A, Simões BM, Clarke RB, Brennan K. A Role for Notch Signalling in Breast Cancer and Endocrine Resistance. Stem Cells Int [Internet]. Hindawi Publishing Corporation; 2016 [cited 2016 Nov 2];2016:2498764. Available from: http://www.ncbi.nlm.nih.gov/pubmed/26880941. 10.1155/2016/2498764 PMC4736972 26880941

[45] Rizzo P, Miao H, D’Souza G, Osipo C, Song LL, Yun J, et al. Cross-talk between notch and the estrogen receptor in breast cancer suggests novel therapeutic approaches. Cancer Res [Internet]. 2008 [cited 2018 Apr 12];68(13):5226–5235. Available from: http://cancerres.aacrjournals.org/cgi/doi/10.1158/0008-5472.CAN-07-5744 10.1158/0008-5472.CAN-07-5744 PMC4445363 18593923

