# Supplementary figures and images for: FKBPL and its peptide derivatives inhibit endocrine therapy resistant cancer stem cells and breast cancer metastasis by downregulating DLL4 and Notch4
Source: BMC Cancer. 2019 Apr 11;19:351. doi: 10.1186/s12885-019-5500-0 (PMC6460676; doi:10.1186/s12885-019-5500-0)

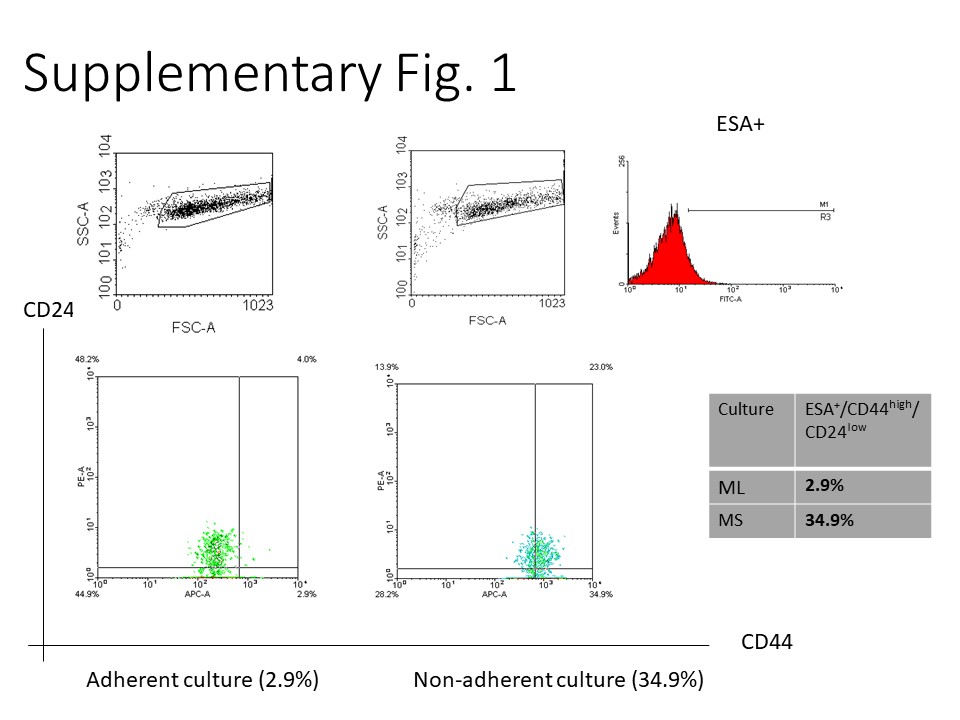

Supplement: Supplementary file 1 — Figure S1. The content of ESA+/CD44+/CD24− cell population within mammosphere culture in MDA-MB-231 cells. (JPG 78 kb) [file 12885_2019_5500_MOESM1_ESM.jpg]

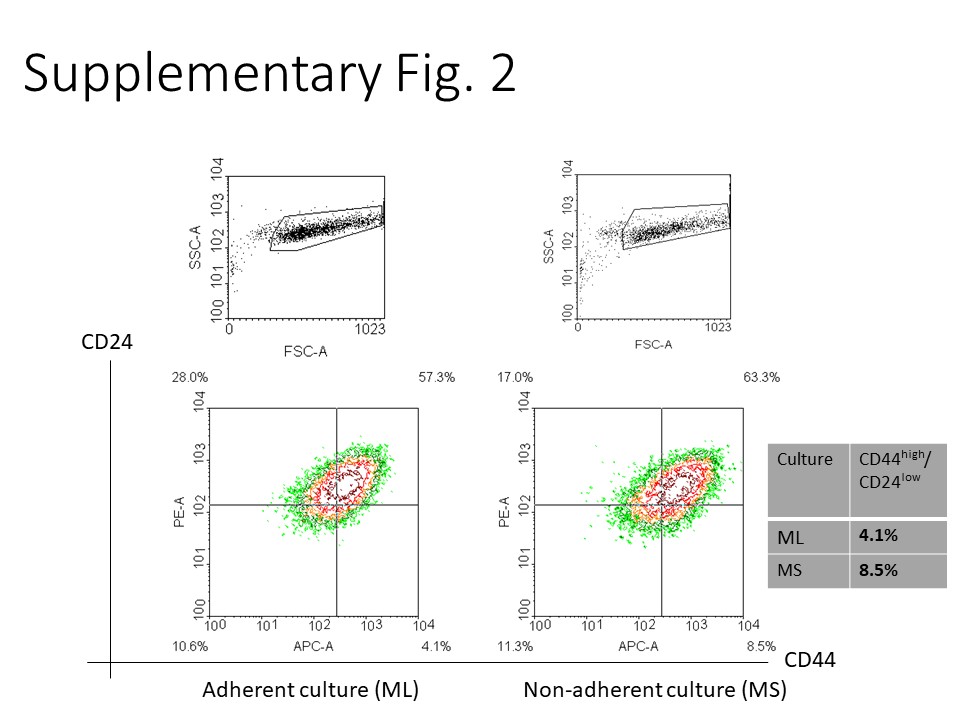

Supplement: Supplementary file 2 — Figure S2. The content of CD44+/CD24− cell population within mammosphere culture in MCF-7 cells. (JPG 89 kb) [file 12885_2019_5500_MOESM2_ESM.jpg]

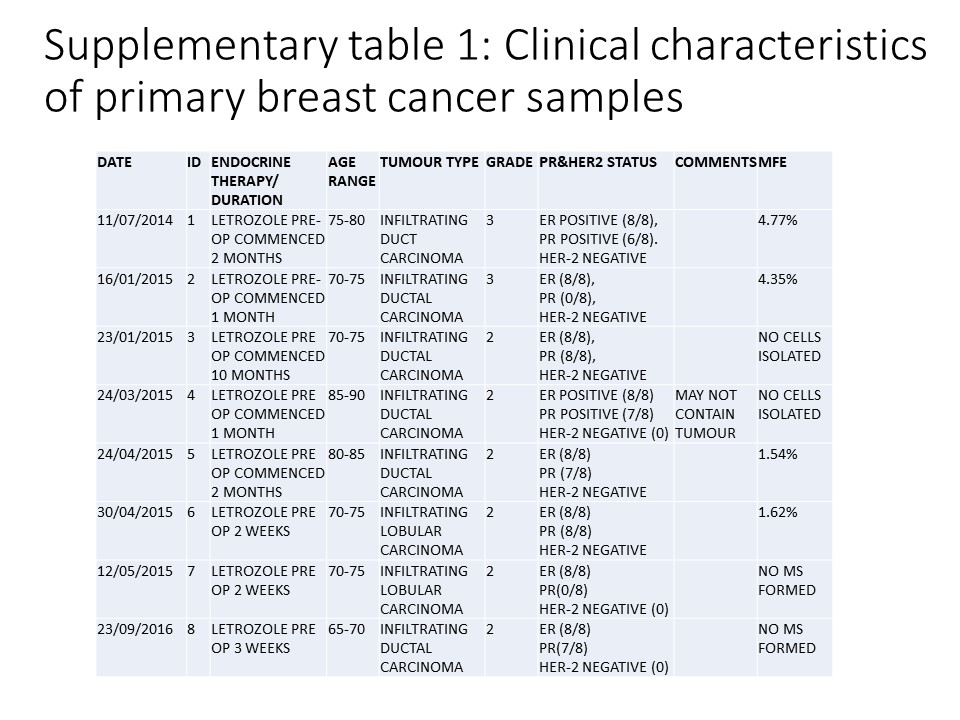

Supplement: Supplementary file 3 — Table S1. The clinical characteristic of primary samples used in the study. (JPG 153 kb) [file 12885_2019_5500_MOESM3_ESM.jpg]

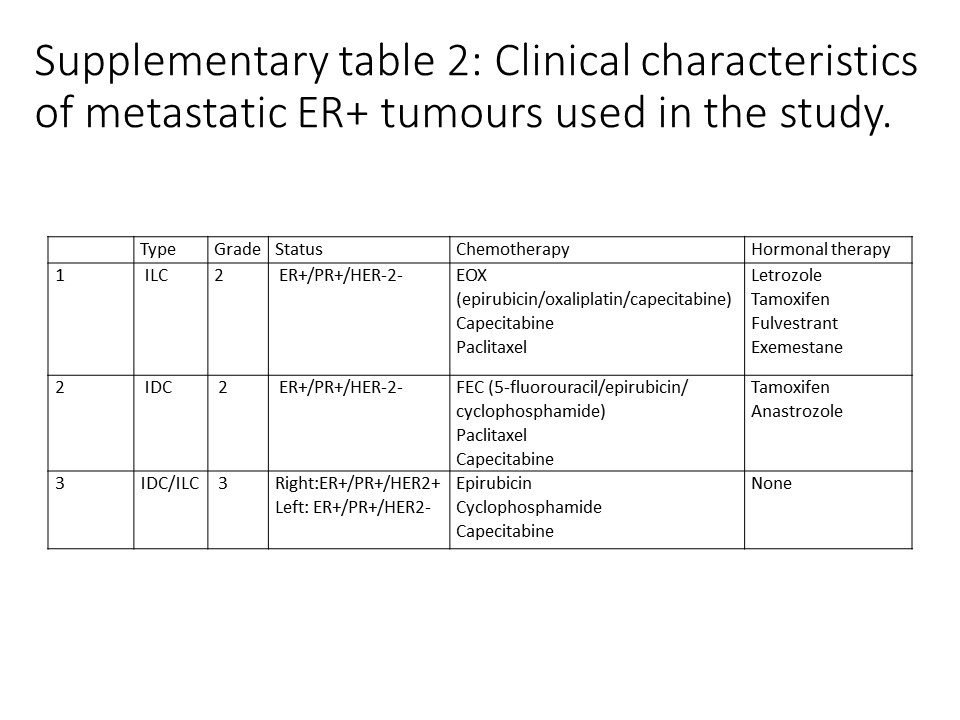

Supplement: Supplementary file 4 — Table S2. The treatment regimens used in metastatic ER+ patient samples. (JPG 97 kb) [file 12885_2019_5500_MOESM4_ESM.jpg]
